# The Slowing Rate of CpG Depletion in SARS-CoV-2 Genomes Is Consistent with Adaptations to the Human Host

**DOI:** 10.1093/molbev/msac029

**Published:** 2022-02-03

**Authors:** Akhil Kumar, Nishank Goyal, Nandhini Saranathan, Sonam Dhamija, Saurabh Saraswat, Manoj B Menon, Perumal Vivekanandan

**Affiliations:** 1 Kusuma School of Biological Sciences, Indian Institute of Technology Delhi, New Delhi, India; 2 Department of Chemical Engineering, Indian Institute of Technology Delhi, New Delhi, India; 3 CSIR-Institute of Genomics and Integrative Biology, New Delhi, India; 4 Academy of Scientific and Innovative Research (AcSIR), Ghaziabad, India

**Keywords:** CpG depletion, SARS-CoV-2, temporal variation, ZAP-binding motif, codon positions, variants of concern

## Abstract

Depletion of CpG dinucleotides in severe acute respiratory syndrome coronavirus-2 (SARS-CoV-2) genomes has been linked to virus evolution, host-switching, virus replication, and innate immune responses. Temporal variations, if any, in the rate of CpG depletion during virus evolution in the host remain poorly understood. Here, we analyzed the CpG content of over 1.4 million full-length SARS-CoV-2 genomes representing over 170 million documented infections during the first 17 months of the pandemic. Our findings suggest that the extent of CpG depletion in SARS-CoV-2 genomes is modest. Interestingly, the rate of CpG depletion is highest during early evolution in humans and it gradually tapers off, almost reaching an equilibrium; this is consistent with adaptations to the human host. Furthermore, within the coding regions, CpG depletion occurs predominantly at codon positions 2-3 and 3-1. Loss of ZAP (Zinc-finger antiviral protein)-binding motifs in SARS-CoV-2 genomes is primarily driven by the loss of the terminal CpG within the motifs. Nonetheless, majority of the CpG depletion in SARS-CoV-2 genomes occurs outside ZAP-binding motifs. SARS-CoV-2 genomes selectively lose CpGs-motifs from a U-rich context; this may help avoid immune recognition by TLR7. SARS-CoV-2 alpha-, beta-, and delta-variants of concern have reduced CpG content compared to sequences from the beginning of the pandemic. In sum, we provide evidence that the rate of CpG depletion in virus genomes is not uniform and it greatly varies over time and during adaptations to the host. This work highlights how temporal variations in selection pressures during virus adaption may impact the rate and the extent of CpG depletion in virus genomes.

## Introduction

Depletion of CpG dinucleotides has been documented in DNA and RNA viruses infecting humans and animals. CpG depletion from DNA viruses has been attributed to deamination of methylated cytosines within CpG dinucleotides (Upadhyay et al. 2013, 2014; Upadhyay and Vivekanandan 2015). We had previously demonstrated a role for host DNA methyltransferases in CpG depletion from DNA viruses (Upadhyay et al. 2013). The Zinc-finger antiviral protein (ZAP) is a host antiviral protein that can selectively bind to CpG-rich viral RNA resulting in their degradation and inhibition of virus replication (Takata et al. 2017; Meagher et al. 2019). ZAP has been recently identified as a potential driver of CpG depletion in RNA viruses (Ficarelli et al. 2020; Nchioua et al. 2020; Xia 2020). Among beta coronaviruses, severe acute respiratory syndrome coronavirus-2 (SARS-CoV-2) has been shown to be the most CpG-depleted virus (Xia 2020). CpG dinucleotide deficiency in SARS-CoV-2 has been studied to understand evolutionary pressures, virus origin, host-switching, virus replication, and evasion of innate immune responses (Nchioua et al. 2020; Pollock et al. 2020; Xia 2020; Di Gioacchino et al. 2021). The adaptive evolution of coronaviruses in bats was associated with CpG depletion (Nchioua et al. 2020; MacLean et al. 2021) before it infected humans. Nonetheless, the extent and the rate of CpG depletion in complete SARS-CoV-2 genomes during their evolution in humans remain poorly understood. Further, although it is well-documented that CpG depletion occurs in several RNA viruses, it is not known whether the rate of CpG depletion remains constant or it varies during virus evolution in the host. In other words, temporal variations, if any, in the rate of CpG depletion during virus evolution and adaptation to the host are not understood. The availability of over 1.4 million complete SARS-CoV-2 genomes representing over 170 million documented cases of human infections in a 17-month period provides a unique opportunity to investigate the temporal evolution of CpG dinucleotides and the evolutionary pressures associated with CpG depletion.

## Results and Discussion

### Modest Depletion of CpGs in SARS-CoV-2 Genomes in the First 17 Months of the Pandemic

**Fig. 1.**
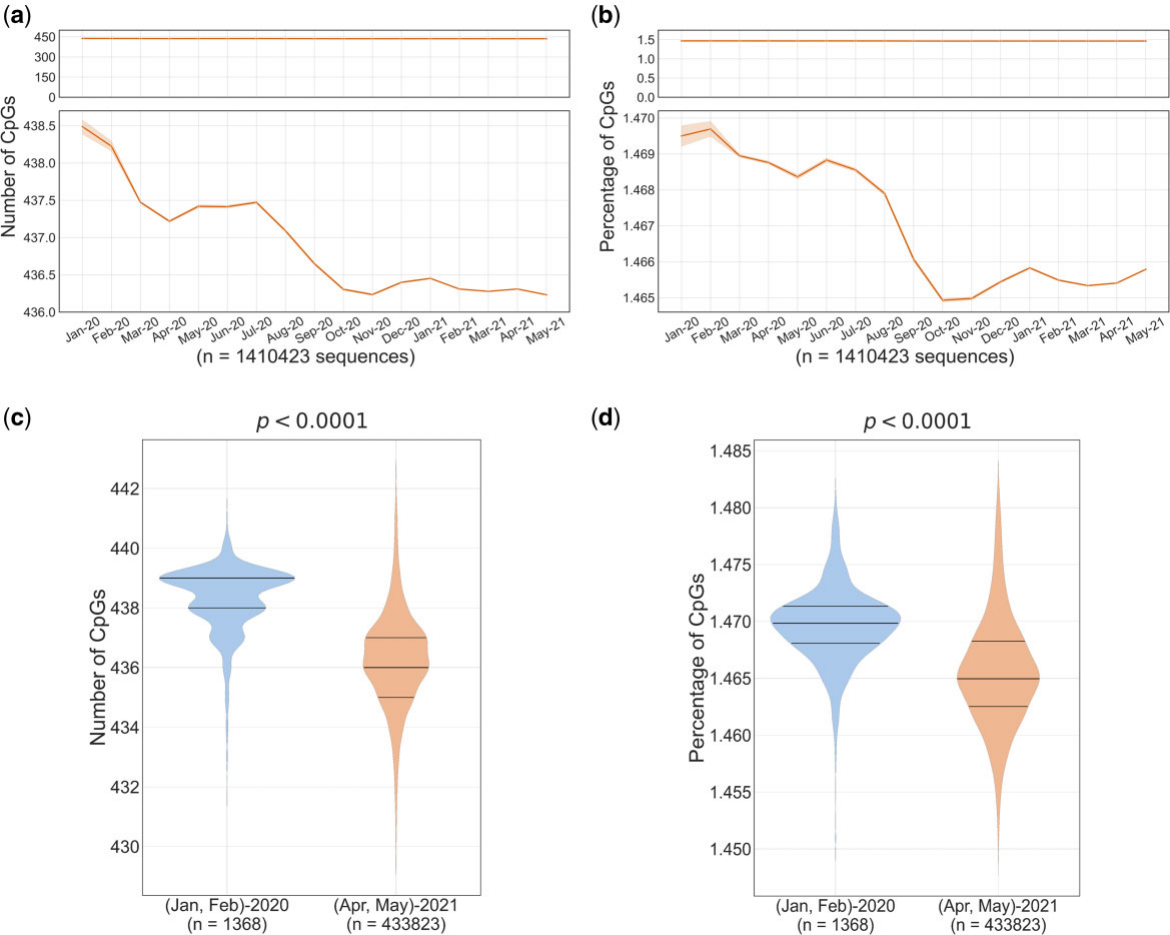
Temporal analysis of CpG content in complete SARS-CoV-2 genomes: full-length sequences of SARS-CoV-2 (*n* = 1,410,423) were grouped month-wise based on the date of sample collection. Line graphs indicate the monthly mean of (*a*) the number of CpGs and (*b*) the percentage of CpGs (normalized to the length of the genome). The line plots in the upper panel show almost a flat line indicating only marginal change in CpG content, whereas the zoomed-in view in the lower panels show modest, but clear trend of CpG depletion. The 95% confidence intervals are represented by the orange bands. Violin plots showing the distribution of (*c*) CpG numbers and (*d*) CpG percentage of the SARS-CoV-2 genomes from samples collected in the first 2 months of the pandemic (i.e., January–February 2020) and those from the last 2 months (i.e., April–May 2021) of the timeline analyzed in this study. The median number of CpGs and the CpG percentage were significantly higher in the samples collected in the first 2 months (January–February 2020) as compared with those in the last 2 months (April–May 2021) (*P* < 0.0001; Mann–Whitney *U* test).

We analyzed the CpG dinucleotide content in full-length SARS-CoV-2 genomes (*n* = 1,410,423) available in GISAID database as on August 24, 2021 (supplementary table 1, Supplementary Material online). We observe that the extent of CpG depletion in SARS-CoV-2 genomes is modest during the first 17 months of the pandemic corresponding to over 170 million documented human infections (fig. 1*a* and *c*). On average, not more than three CpGs were lost from full-length SARS-CoV-2 genomes starting from January 2020 to May 2021 (fig. 1*a* and *c*). We also measured the CpG content of SARS-CoV-2 genomes as an absolute percentage of all dinucleotides (CpG percentage) after correcting for minor differences in length of whole genome sequences available. Both CpG numbers and CpG percentage confirm modest depletion of CpG dinucleotides (fig. 1*a*–*d*) in the first 17 months of the pandemic. We believe that the extent of CpG depletion during the evolution of SARS-CoV-2 in humans is modest considering that: 1) over 7,000 mutations have been described (Chen et al. 2021; Wang et al. 2021) during this period, 2) a plethora of host factors including APOBEC (apolipoprotein B mRNA-editing catalytic polypeptide) editing (Mourier et al. 2021) and binding of ZAP to SARS-CoV-2 RNA (Kamel et al. 2021) have been linked to CpG depletion (Nchioua et al. 2020; Mourier et al. 2021). There are no yardsticks available to compare the temporal loss of CpGs in virus genomes. The best and perhaps the only example of a study investigating the loss of CpGs in full-length virus genomes over time was done by Greenbaum et al. (2008), on influenza viruses, which are single-stranded negative sense RNA viruses that lack proof reading ability and contain segmented genomes capable of reassortment. In their study, about 2,300 influenza A virus sequences from samples collected over several decades were investigated. Their data suggest that the CpG content may fluctuate over 5% within the same year for some subtypes of influenza A virus. In contrast, in our study, we find that the extent of CpG loss in SARS-CoV-2 genomes is far less than 1% (i.e., about 2-3 of 439 CpGs are lost) in 17 months of evolution in human hosts (fig. 1). Although differences in CpG content along different coding and noncoding regions of SARS-CoV-2 have been documented (Di Gioacchino et al. 2021), our goal was to perform temporal analysis of CpG depletion at the genome scale.

The analysis of CpG content in sequences from samples collected at the beginning of the pandemic (i.e., January and February 2020; *n* = 1,368) and those from April and May 2021 (*n* = 433,823) reconfirms the modest loss of CpGs from SARS-CoV-2 genomes during this period (fig. 1*c* and *d*). Notably, despite the extent of genetic diversity documented in SARS-CoV-2 genomes, the genome-wide CpG content appears to be governed stringently by evolutionary forces as evidenced by the tight range (fig. 1*a*–*d*). This is particularly interesting considering that SARS-CoV-2 genome, despite its already low CpG content, has been recently shown to be susceptible to the antiviral activity of ZAP which is mediated by CpG binding (Nchioua et al. 2020). Although the possible reasons for modest CpG depletion from SARS-CoV-2 genomes during evolution in humans merit further investigation, we speculate that combinations of mutations that provide significant survival-, replication-, or infectivity-advantages may be preferentially selected over CpG-depleted variants that may provide protection from ZAP-mediated restriction. It is also possible that SARS-CoV-2 genomes are already extensively CpG depleted (Digard et al. 2020; MacLean et al. 2021) and further loss may be potentially deleterious.

### Rate of CpG Depletion Is Highest during Early Evolution in Humans

Mutation rates for a given virus are calculated per site per round of replication (Duffy et al. 2008). In the ongoing pandemic, the number of infections (or number of infected individuals) is a surrogate for the number of replication cycles. In other words, the opportunity to generate mutations in the SARS-CoV-2 genomes should increase with the number of infections. However, this does not mean that substitution rates (i.e., rate at which mutations become fixed at a population level; substitutions/site/year) at population level increase with the number of infections. In fact, substitution rates may gradually taper off during virus evolution in a host (Scholle et al. 2013). Although mutations leading to the loss of CpGs may help the virus evade host innate immune responses, they can also be deleterious or neutral. Further, mutation rates respond to natural selection and can vary during the course of virus evolution (Duffy 2018; Peck and Lauring 2018).

**Fig. 2.**
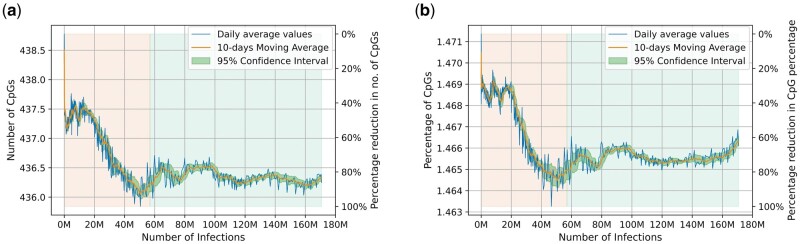
CpG content in SARS-CoV-2 genomes has stabilized with increasing number of infections: Number of SARS-CoV-2 sequences available on each day was determined along with the cumulative number of documented infections until the given day. *Y* axis on the right indicates the extent of reduction between minimum and maximum value of CpG metrics. Grids are colored orange and green to demarcate the total number of documented infections into the first one-third and the subsequent two-thirds, respectively. The line graphs indicate that majority (∼>85%) of the CpG depletion in the SARS-CoV-2 genomes occurred well before the first one-third (orange grid) of documented infections (i.e., 56.92 million infections corresponding to November 2020). The CpG content appears to be stabilized in the green part (i.e., during the subsequent 113.87 million infections) indicated by almost constant values.

The number of full-length SARS-CoV-2 sequences available in GISAID has a linear relationship with the number of documented infections (supplementary fig. 1*a*, Supplementary Material online), confirming that our analysis is not biased by disproportionate number of sequences compared with the number of documented cases at any given point of time. The number of SARS-CoV-2 infections increased exponentially during the study period (supplementary fig. 1*b*, Supplementary Material online). The epidemic rate (i.e., rate at which transmissions occur) may influence virus evolutionary dynamics (Berry et al. 2007). In addition, it is also possible that the selection pressures shaping virus evolution may have varied during this 17-month period (January 2020 to May 2021). We therefore sought to understand how the number of human infections impacts CpG depletion from the SARS-CoV-2 genomes. For this purpose, we plotted CpG content (CpG numbers and CpG percentage) of full-length SARS-CoV-2 genomes against number of infections for each day of the infection timeline analyzed (fig. 2*a* and *b*). The full-length sequences analyzed in this study represent over 170 million documented cases of SARS-CoV-2 infections across the world. Interestingly, about 85% of CpG depletion occurred in SARS-CoV-2 genomes during the first one-third of documented infections (∼57 million; orange grid in fig. 2) (fig. 2*a* and *b*). In contrast, there is little or no CpG depletion in the genomes available from the subsequent 114 million cases (green grid; fig. 2*a* and *b*). This finding suggests that the rate of CpG depletion is highest during early evolution of SARS-CoV-2 in humans, gradually tapering off with increasing number of infections.

When a virus is well-adapted to the host, the selection pressures will be negligible (Peck and Lauring 2018). We observed an initial rapid decline of CpGs followed by slower rates of decline with increasing number of infections. This is consistent with progressive adaptation of SARS-CoV-2 in humans with increasing number of infections leading to reduced selection pressure for CpG depletion. Furthermore, our data suggest that CpG content in SARS-CoV-2 genomes has stabilized or almost reached an equilibrium when 40–50 million infections were documented world-wide (∼9–11 months from the onset of the pandemic). This finding is in keeping with a previous report suggesting the lack of major changes in the CpG content in influenza B virus, a well-adapted human pathogen (Greenbaum et al. 2008). In addition, since SARS-CoV-2 genomes represent human coronaviruses with the lowest CpG content (Digard et al. 2020), the observed stabilization of CpG content in our study may reflect the limited evolutionary space available for CpG depletion. However, our findings that the CpG content of SARS-CoV-2 remains stable for over 7 months (i.e., October 2020 to May 2021, corresponding to over 110–120 million documented human infections) do not by any means rule out the possibility of additional CpG depletion in future.

Another possible explanation for the slowing CpG depletion from SARS-CoV-2 genomes with increasing number of cases comes from a previous report on HIV-1 evolution. A higher epidemic rate (i.e., rate of spread of infections) has been associated with slower evolutionary rates for HIV-1 (Berry et al. 2007). The repeated transmission of HIV-1 from an individual in the early stages of infection reduces the window in which immune selection pressures can impact virus evolution. Although limited information is available on the extent of multiple transmissions during the early stages of SARS-CoV-2 infections, the exponential increase in number of infections along with increased transmission potential for some variants suggest increased transmission events. It is therefore possible that variations in transmission dynamics may contribute to the slowing and stabilization of CpG depletion from SARS-CoV-2 genome.

**Fig. 3.**
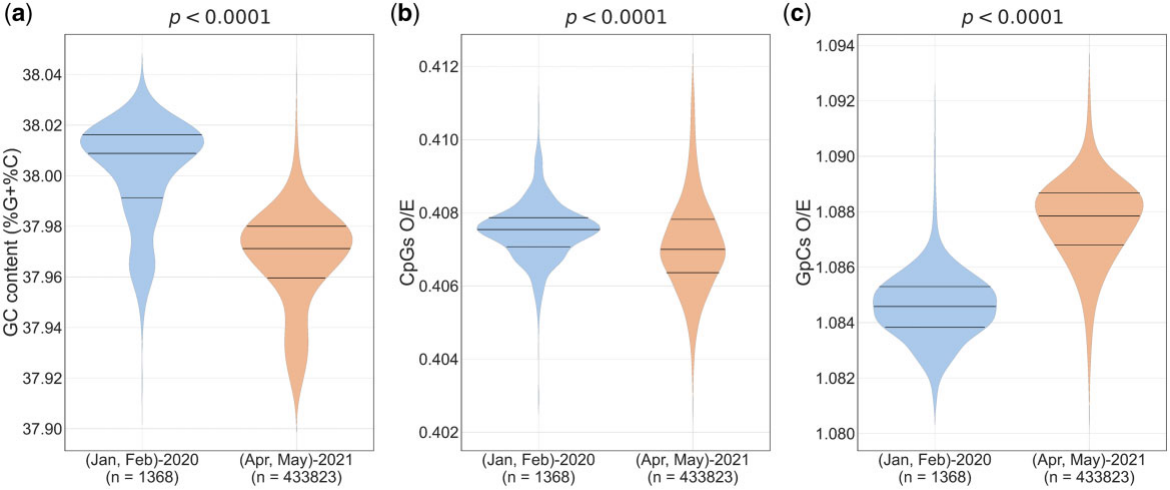
CpG depletion in SARS-CoV-2 is not linked to the loss of constituent mononucleotides: violin plots showing the distribution of (*a*) GC content, (*b*) CpGs *O*/*E* ratios, and (*c*) GpCs *O*/*E* ratios among SARS-CoV-2 sequences collected in the first 2 months (January–February 2020) and last 2 months (April–May 2021) of the study period. Over the course of the pandemic, a statistically significant decrease in GC content was observed. Contrasting trends in the relative abundance of CpG and GpC dinucleotides were noted; CpG *O*/*E* ratios decreased, whereas GpC *O*/*E* ratios increased during the 17-month period. The Mann–Whitney *U* test was used to compare the medians. *O*/*E*, observed/expected.

APOBECs have been linked to C-to-U deamination of bases in single-stranded RNA (i.e., the +ssRNA and also the −ssRNA that serves as the replication intermediate). Theoretically, APOBEC-mediated editing can lead to C-to-U (or) G-to-A changes in SARS-CoV-2 genomes (Di Giorgio et al. 2020). Nonetheless, no evidence of increased G-to-A mutations was found in SARS-CoV-2 genomes (Simmonds 2020; Di Giorgio et al. 2020). APOBEC-editing may lead to the depletion of C residues from the SARS-CoV-2 genomes. We mapped the changes in the percentage of the four mononucleotides (A, C, G, and U) in the SARS-CoV-2 genomes with time (supplementary fig. 2*a*–*d*, Supplementary Material online). The percentage of Cs decreased with increasing number of infections, whereas the percentage of Us increased. Importantly, the GC content (i.e., G% + C% or G + C content) of the SARS-CoV-2 genome clearly shows a downward trend with time (supplementary fig. 2*e*, Supplementary Material online). Hence, we asked the question if the loss of CpGs from the SARS-CoV-2 genomes is merely a result of the loss of constituent mononucleotides (i.e., Cs and Gs) or is there a role for specific selection pressures on CpG depletion? The GC content of SARS-CoV-2 genomes were significantly lower in samples collected in April and May 2021 as compared with those collected in the beginning of the pandemic (fig. 3*a*). To investigate if the depletion of CpG dinucleotide content in SARS-CoV-2 is linked to the reduction in GC content, we calculated the relative abundance of CpG dinucleotides (i.e., CpG dinucleotide content normalized to the numbers of the constituent mononucleotides; this is referred to as *O*/*E* [observed/expected] ratios). If the CpG depletion in SARS-CoV-2 is merely a reflection of loss of Cs and Gs from the genome, one would expect that during the 17 months of evolution, CpG *O*/*E* ratios will either: 1) almost remain constant (i.e., the number of CpGs lost is proportional to the loss of Cs and Gs) or 2) increase (i.e., if the extent of Cs and Gs lost is more pronounced than extent of CpGs lost from the genome). Nonetheless, the CpG *O*/*E* ratios were significantly lower in sequences from April and May 2021 compared with those from the beginning of the pandemic (fig. 3*b*), indicating that the depletion of CpGs is not necessarily linked to the loss of the constituent mononucleotides.

To further investigate this, we examined GpC dinucleotides. GpC dinucleotides are made up of the same constituent mononucleotides as CpGs. Unlike CpG *O*/*E* ratios, we found that GpC *O*/*E* ratios are higher for sequences from samples collected in the last 2 months as (April and May 2021) compared with those from the beginning of the pandemic (fig. 3*b* and *c*). The decrease in CpGs *O*/*E* ratios despite a loss of GC content and an increase in GpC *O*/*E* ratios during this period (fig. 3*a*–*c*), reiterate that the depletion of CpGs from the SARS-CoV-2 genomes is not linked to the loss of the constituent mononucleotides. Although these findings do not rule out a role for APOBECs in the loss of CpGs from SARS-CoV-2, they confirm a definitive role for specific selection pressure contributing to the avoidance of CpGs over and above mutations/host enzyme-mediated editing leading to the loss of GC content.

### Increased CpG Depletion from Codon Positions 2-3 and 3-1 Positions in SARS-CoV-2 Genomes

**Fig. 4.**
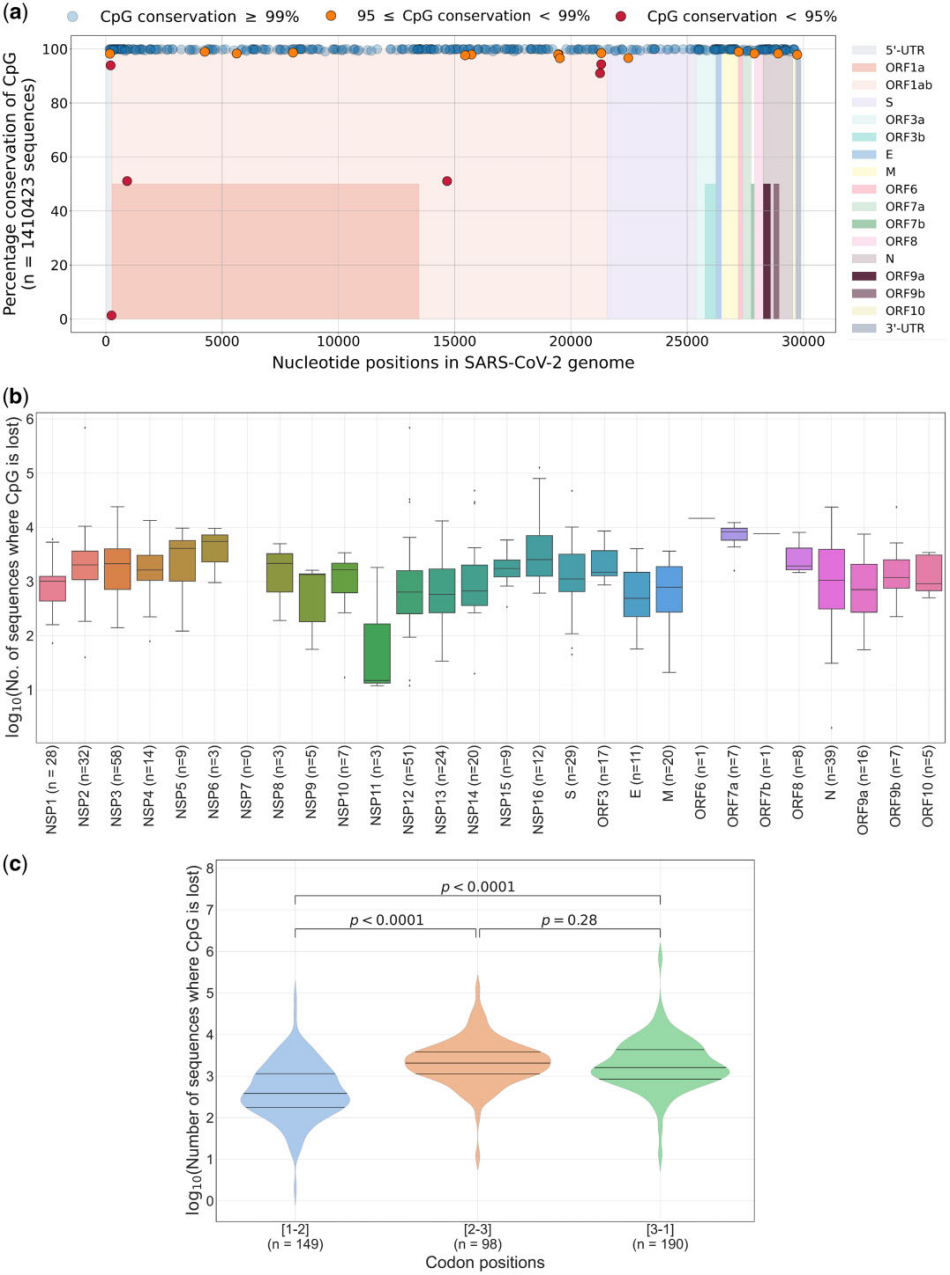
The extent of CpG depletion across genes and codon positions: (*a*) Graph showing the location of CpG dinucleotides in the SARS-CoV-2 (nucleotide positions are based on the WIV04 reference sequence) and their conservation across 1,410,423 complete SARS-CoV-2 genomes. Different colors are used along the *x* axis to indicate genes/ORFs. For overlapping ORFs, segments are colored to half the height. The graph shows that majority of the CpG dinucleotides are conserved in over 99% of the sequences analyzed. (*b*) Gene-wise analysis of CpG loss: the number of CpGs within each gene is indicated on the *x* axis. Box-and-whiskers plots show the gene-wise distribution of sequences that lost CpG dinucleotide(s). Outliers are shown as black dots. The extent of CpG depletion varies greatly within and across genes. (*c*) CpGs within coding regions of SARS-CoV-2 are grouped on the basis of codon positions. The loss of CpGs at codon positions 2-3 and 3-1 is more pronounced than that at codon position 1-2. The Mann–Whitney *U* test was used to compare the medians in the violin plots. NSP, nonstructural protein; ORF, open reading frame.

To visualize if the CpG depletion in SARS-CoV-2 genomes occurs at specific genomic locations, we plotted the conservation of each CpG site (mapping was done with the SARS-CoV-2 WIV04 reference sequence; see Materials and Methods for details) along the length of the SARS-CoV-2 genome (fig. 4*a*). CpG depletion at majority of the CpG sites was observed in less than 1% of the sequences analyzed (*n* = 1,410,423) (fig. 4*a*). A total of 15 CpG sites were lost from 1% to 5% of sequences and six CpG sites were lost from >5% of sequences (fig. 4*a*).

We then plotted the distribution of CpGs lost within each gene (fig. 4*b*). We observed variations in the number of CpG sites within a gene and the extent of CpG depletion within and across genes (fig. 4*b*). The number of CpG dinucleotides vary from 0 to 51 among different genes (or ORFs) and the extent of CpG depletion varies over a range of three to four orders of magnitude both within and across genes (or ORFs) in SARS-CoV-2 genomes (fig. 4*b*). Although this finding reiterates heterogeneity in both distribution of CpG dinucleotides and the extent of their depletion across the SARS-CoV-2 genomes that has been reported previously (Di Gioacchino et al. 2021; Rice et al. 2021), we could not make further conclusions about the differences in the extent of CpG loss from specific genes/transcripts of the SARS-CoV-2 genome.

Codon usage bias explains, at least in part, the loss of CpGs from virus genomes (Belalov and Lukashev 2013). We wanted to understand if the extent of CpG depletion from the coding region of SARS-CoV-2 is linked to the location of the CpG at specific codon positions 1-2 (i.e., CGN) or 2-3 (i.e., NCG) or 3-1 (i.e., NNC-GNN). For this purpose, we mapped the number of sequences that lost CpGs from coding regions to their position within the codon (fig. 4*c*). Notably, the median number of sequences that lost a CpG dinucleotide from codon positions 2-3 and 3-1 were significantly higher as compared with those from codon position 1-2 (fig. 4*c*). Differences in evolutionary constraints across the three codon positions are well-documented (Belalov and Lukashev 2013). The third position in the codon is referred to as the wobble position and it may allow for nucleotide substitutions without changes in the amino acid. The increased depletion of CpGs from codon positions 2-3 and 3-1 compared with that in codon position 1-2 in the SARS-CoV-2 genomes may be due to the presence of the wobble position in both 2-3 and 3-1 positions; CpG depletion at codon position 1-2 is constrained by accompanying amino acid changes. CpG content at codon position 2-3 has a stronger influence on codon usage bias as compared with that at position 3-1 (Belalov and Lukashev 2013). However, stronger suppression of CpGs at codon position 3-1 than at 2-3 has been documented for some viruses infecting humans (Belalov and Lukashev 2013). For SARS-CoV-2 genomes, we found that the extent of CpG depletion from codon positions 2-3 and 3-1 was comparable (fig. 4*c*). Increasing the CpG content within codons without altering the amino acid sequence of poliovirus capsid proteins led to a reduction in virion secretion and infectivity (Burns et al. 2009). We believe that the significant reduction of CpG content from codon positions 2-3 and 3-1 in SARS-CoV-2 genomes may have implications on virus replication and transmission.

### The Extent of CpG Loss within and outside ZAP-Binding Motifs Is Comparable in SARS-CoV-2 Genomes

ZAP can restrict viral RNA by binding to CpG-rich regions in virus genomes (Takata et al. 2017) with the help of several cofactors including TRIM25 (a ubiquitin ligase) and KHNYN (a KH-domain containing endonuclease) (Zheng et al. 2017; Ficarelli et al. 2019). Recently, optimal ZAP-binding motifs with a terminal CpG dinucleotide and additional G and C sites separated by four to eight nucleotides (spacers) were identified (i.e., C(n_*m*_)G(n)CG, where *m* = 4/5/6/7/8) (Luo et al. 2020).

**Fig. 5.**
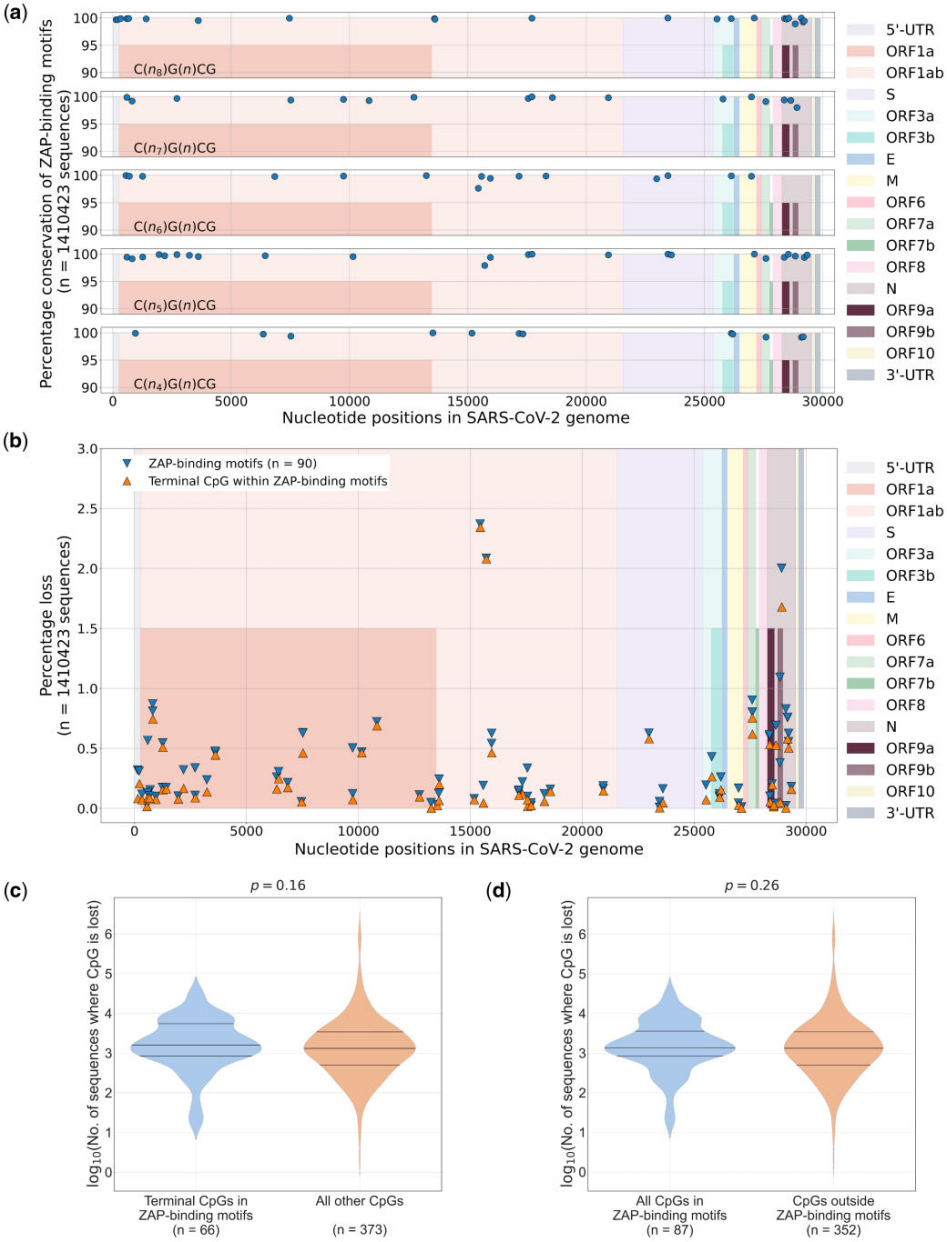
Loss of CpG dinucleotides and ZAP-binding motifs in SARS-CoV-2 genomes: (*a*) Graphs showing the location and conservation of ZAP-binding motifs in the SARS-CoV-2 genome. Genes in the SARS-CoV-2 genome are indicated in different colors. (*b*) All ZAP-binding motifs end with a terminal CpG dinucleotide. The percentage of sequences that lost ZAP-binding motifs and the terminal CpG within the motifs are plotted. The loss of ZAP-binding motifs from the SARS-CoV-2 genomes is primarily associated with the loss of the terminal CpG within motifs. (*c*) The number of sequences that lost CpG dinucleotides in the SARS-CoV-2 genome were analyzed. The extent of CpG depletion from the terminal CpG site within the ZAP-binding motif is comparable to that from the other CpG sites (i.e., all CpGs other than the terminal CpG site within ZAP-binding motifs) in the SARS-CoV-2 genome. Since ZAP-binding motifs may be overlapping, only nonoverlapping or unique CpG sites are considered. (*d*) The loss of CpGs from all the CpG sites in ZAP-binding motifs (includes the terminal CpG and all other CpGs within ZAP-binding motifs) is comparable to that from all the CpG sites outside ZAP-binding motifs in the SARS-CoV-2 genomes. Since ZAP-binding motifs may be overlapping, only nonoverlapping or unique CpG sites are considered for the violin plots. The Mann–Whitney *U* test was used to compare the medians in the violin plots. ZAP, zinc-finger antiviral protein.

To understand the association between the loss of CpGs and ZAP-binding motifs, we analyzed the loss of optimal ZAP-binding motifs (please see Materials and Methods for details on the five ZAP-binding motifs analyzed) from SARS-CoV-2 genomes. Among the 90 ZAP-binding motifs we identified in the SARS-CoV-2 reference sequence, 86 ZAP-binding motifs were over 99% conserved among the 1,410,423 sequences analyzed (fig. 5*a*). Only four ZAP-binding motifs were lost in over 1% of the sequences analyzed; two of them correspond to the N gene and the overlapping ORF 9 b and the other two motifs are part of RNA dependent RNA polymerase encoding region of SARS-CoV-2 (supplementary table 2, Supplementary Material online). ZAP-mediated pressure has been implicated in the loss of CpGs from SARS-CoV-2 genomes, particularly from the N gene (Di Gioacchino et al. 2021).

Optimal ZAP-binding motifs may vary from 9 to 13 nucleotides in length (Luo et al. 2020). ZAP-binding motifs may contain CpGs other than the terminal CpG. The loss of the terminal CpG from the ZAP-binding motif will lead to the loss of the motif. However, the loss of other CpGs within the motif may not necessarily lead to the loss of ZAP-binding motifs. Therefore, we sought to investigate the association between the loss of a ZAP-binding motif and the loss of the terminal CpG site. With a few exceptions, the proportion of sequences that lost the terminal CpG from the ZAP-binding motif closely mirrors the proportion of sequences that lost a ZAP-binding motif (fig. 5*b*), suggesting that the loss of ZAP-binding motifs in SARS-CoV-2 genomes is primarily driven by the loss of the terminal CpG dinucleotide.

We then asked the question whether the depletion of CpG dinucleotides in the SARS-CoV-2 genome is driven by the loss of CpGs from within ZAP-binding motifs. For this purpose, we compared the loss of CpGs from within ZAP-binding motifs with that from the rest of the SARS-CoV-2 genome. As ZAP-binding motifs may be overlapping, the same CpG position may be shared by two or more motifs. Therefore, only nonoverlapping or unique CpG sites were considered for this analysis (see Materials and Methods for details). Interestingly, the median number of sequences that had lost a terminal CpG or any CpG from within a ZAP-binding motif was comparable to those that had lost a CpG from regions that lie outside of ZAP-binding motifs in the SARS-CoV-2 genome (fig. 5*c* and *d*). In other words, the extent of CpG depletion in the SARS-CoV-2 genome is comparable from locations within ZAP-binding motifs and the rest of the genome. Taken together, our data suggest that: 1) the loss of ZAP-binding motifs from SARS-CoV-2 genomes is primarily driven by the loss of the terminal CpG site within the motif and 2) the extent of CpG depletion for sites within and outside ZAP-binding motifs in SARS-CoV-2 genomes is comparable. Our results also suggests that the negative selection of CpGs from ZAP-binding motifs explains only a fraction of the CpG depletion in SARS-CoV-2 genomes (since nearly 80% of the CpG sites in the genome are outside ZAP-binding motifs). Although we cannot rule out the existence of yet unknown ZAP-binding motifs, our findings highlight a major role for other evolutionary forces that may contribute to CpG depletion apart from ZAP.

### SARS-CoV-2 Genomes Selectively Lose CpG Motifs That Occur in a U-Rich Context

**Fig. 6.**
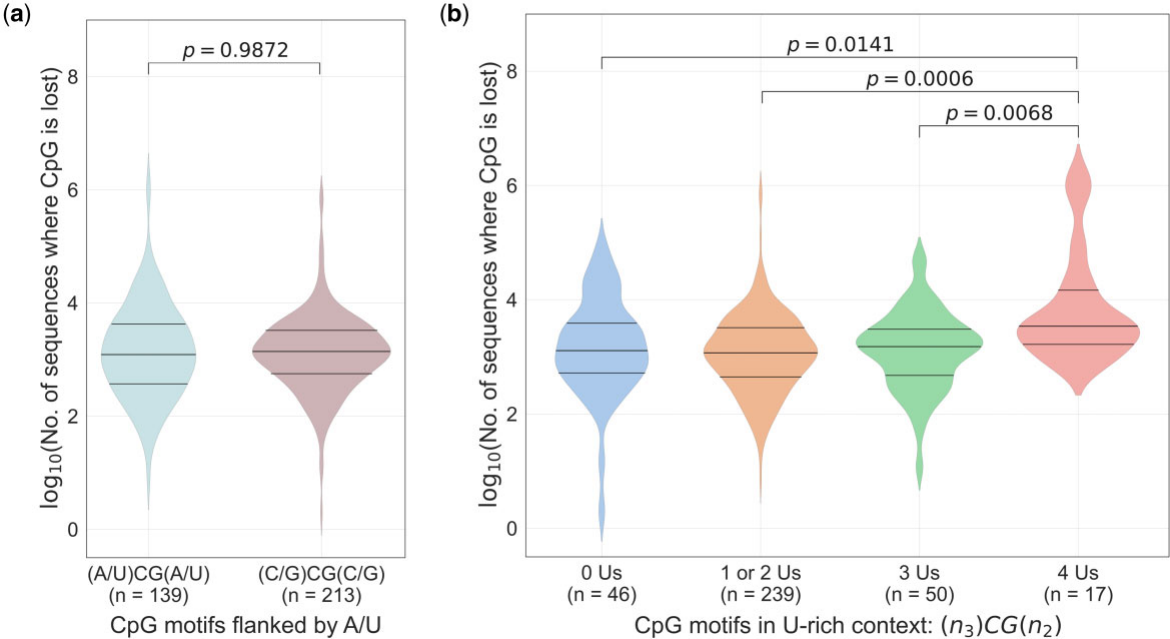
Selective loss of CpG motifs from a U-rich context in SARS-CoV-2 genomes to avoid immune recognition by TLR7: (*a*) The number of sequences that lost CpG motifs in an A/U-rich context was analyzed. The extent of CpG motif depletion from an A/U-rich context was comparable to that from the other CpG sites (i.e., non-A/U-rich context). (*b*) The selective loss of CpG motifs from SARS-CoV-2 genomes in a U-rich context (i.e., 4Us in the motif NNN-CG-NN) as compared with CpG motifs that are flanked by fewer Us (i.e., 1 to 3Us or no Us). There were no CpG motifs that were flanked by 5Us. Only CpGs outside ZAP-binding motifs (*n* = 352) were considered for this analysis (see Materials and Methods for details). The Mann–Whitney *U* test was used to compare the medians. TLR7, toll-like receptor 7.

Greenbaum et al. (2009) demonstrated that the loss of CpG motifs from an A/U-rich context (i.e., (A/U)CG(A/U)) from H1N1 influenza genomes over time. Subsequently, it was demonstrated that the U-content of bases flanking (three bases at the 5` end and two bases at the 3` end) CpG motifs was linked to the ability to induce interferon-α (IFN-α) from plasmacytoid dendritic cells (Jimenez-Baranda et al. 2011) through activation of Toll-like receptor 7 (TLR7). Specifically, CpG motifs flanked by 4Us (e.g., UUU-CG-AU) were able to induce significantly higher levels of IFN-α as compared with CpG motifs with 1U or 2Us (e.g., AAA-CG-AU or AAU-CG-AU). Together, these findings explain the selective loss of CpGs in an A/U-rich context from influenza virus genomes. Therefore, for CpG motifs that occurred outside the ZAP-binding motifs, we investigated if the loss of CpGs occurred specifically in a A/U-rich context. Unlike in H1N1 influenza genomes, we found that the proportion of sequences that lost CpG motifs which are flanked by A/U (i.e., (A/U)CG(A/U)) were comparable to those that lost CpG motifs in a non-(A/U)-rich context (i.e., (C/G)CG(C/G)) (fig. 6*a*). Interestingly, the proportion of SARS-CoV-2 sequences that lost CpG motifs flanked by 4Us (i.e., 4Us in the motif NNN-CG-NN) were significantly higher than those that lost CpG motifs flanked by 1 to 3Us or no Us (fig. 6*b*). This finding provides evidence that during evolution in humans, SARS-CoV-2 genomes selectively lose CpG motifs that occur in a U-rich context to avoid immune recognition by TLR7.

### Most SARS-CoV-2 Variants of Concern Have Lower CpG Content Compared with Sequences from the Beginning of the Pandemic

**Fig. 7.**
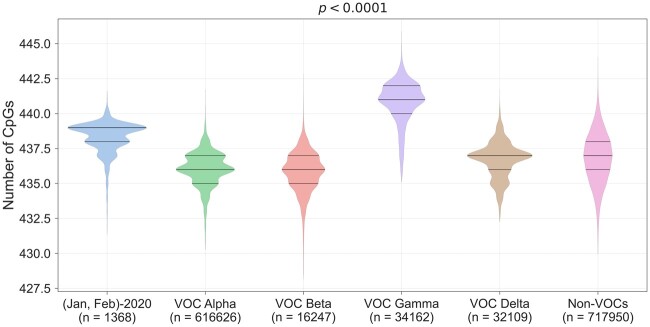
CpG dinucleotide content of SARS-CoV-2 variants of concern (VOCs): the distribution of CpG content in VOCs are plotted. The CpG content for sequences from the beginning of the pandemic (January and February 2020) are also plotted to facilitate comparison. The alpha-, beta-, delta-variants, and all sequences from non-VOCs (i.e., all sequences that are not classified as VOCs) had lower CpG content compared with that in sequences from the beginning of the pandemic. The gamma variant was an outlier with higher CpG content than other variants, non-VOCs, and the sequences from the beginning of the pandemic (pair-wise statistical analysis is provided in supplementary table 4, Supplementary Material online). The medians were compared using a Kruskal–Wallis *H* test. VOC, variant of concern.

Until May 2021, the World Health Organization had identified four variants of concern (VOCs) that have the potential to cause major public health challenges. The SARS-CoV-2 VOCs may have increased transmissibility, evade natural or vaccine-induced immune response, and may be associated with increased pathogenicity (Otto et al. 2021). Each of the VOCs is defined by lineage-specific mutations. The full-length SARS-CoV-2 sequences analyzed were classified into alpha-, beta-, gamma-, and delta-variant; the rest of the sequences that are not classified as a VOC (as in GISAID; supplementary table 3, Supplementary Material online) are referred as “Non-VOCs.” The CpG content of the alpha-, beta-, delta-variants, and non-VOCs were significantly lower as compared with the sequences from the beginning of the pandemic (January–February 2020) (fig. 7 and supplementary table 4, Supplementary Material online). A recent report on the antiviral activity of ZAP on SARS-CoV-2 indicates that the alpha variant (with reduced CpG content compared with the sequences from the beginning of the pandemic) retains sensitivity to ZAP (Kmiec et al. 2021).

Of note, the CpG content of the gamma variant was higher as compared with that in sequences from the beginning of the pandemic, all other VOCs and non-VOC. Since the gamma variant had higher CpG content, we wanted to understand if this could be a result of gamma variant-defining mutations that lead to an increase in CpG content (i.e., by creating a new CpG). On analyzing mutations associated with the alpha- and beta-variant, we found that none of them resulted in a loss or a gain of CpGs. However, mutations associated with the delta variant lead to the gain of two CpGs (T22917G and C23604G) and the loss of one CpG (G15451A). Interestingly, the gamma variant-defining mutations did not lead to a loss of CpGs but were associated with the gain of two CpGs (A22812C and C28512G), thus explaining, at least in part, the higher CpG content of this variant. Our findings on the CpG content of SARS-CoV-2 VOCs suggests that: 1) the CpG content may vary across VOCs, but all variants except for the gamma variant have lower CpG content compared with sequences from the beginning of the pandemic, 2) some of the VOC-lineage defining mutations may lead to a net gain in CpG content. Although CpG depletion from SARS-CoV-2 genomes may help the virus better survive ZAP-mediated restriction and facilitate optimal codon usage, the retention of mutations leading to CpG gain in the gamma variant sheds light on the delicate balance among the evolutionary forces that regulate CpG content in virus genomes. Importantly, our findings suggest that mutations resulting in the gain of new CpGs may be retained if they confer advantages such as increased transmissibility or reducing the efficacy of neutralizing antibodies. Clearly, these advantages outweigh the benefits provided by CpG deselection.

Although the gamma variant represented only a small fraction of the sequences analyzed (<2.5%), since this variant was an outlier with higher CpG content, we wanted to analyze if the high CpG content of this variant could mask the depletion of CpGs in other variants/non-VOC. For this purpose, we plotted the CpG content of the sequences with (all sequences including the gamma variant) and without the gamma variant (all sequences after excluding the gamma variant) over the 17-month period (supplementary fig. 3, Supplementary Material online). It is evident that despite the high CpG content, the gamma variant does not affect any of the conclusions on the extent of CpG depletion in early evolution or the subsequent stabilization of CpG content in SARS-CoV-2 genomes (supplementary fig. 3, Supplementary Material online).

We assessed the association, if any, between CpG stabilization in SARS-CoV-2 genomes and the emergence of VOCs. Our findings indicate that the CpG numbers in SARS-CoV-2 genomes begin to stabilize or reach an equilibrium when about 40 million infections were documented (fig. 2*a*); this period corresponds to September–October 2020. Interestingly, all the four VOCs were first detected during this period (i.e., September–October 2020) as described in VOC reports at PANGO Lineages server (O’Toole et al. 2021). In our data set, we had less than 300 full-length sequences belonging to all the VOCs at the end of October 2020. In other words, our data suggest that the emergence of all the four VOCs coincides with the period corresponding to the slowing and stabilization of CpGs from SARS-CoV-2 genomes. It is evident that VOCs had no role in the initial rapid depletion of CpGs from SARS-CoV-2 genomes. We have two speculative, but intriguing explanations based on the coincidence of the timelines for the emergence of VOCs and the slowing of CpG depletion in SARS-CoV-2 genomes. First, it is possible that the emergence of VOCs is defined by a specific combination of mutations that confer several advantages to the virus including transmissibility, fitness, and reduced neutralization may obliterate the need for further CpG depletion. Second, it is also possible that the depletion of CpG content below a critical threshold is a prerequisite to facilitate the emergence of VOCs. Although both the explanations are no more than speculations, the coincidence of timelines for the emergence of all the VOCs and the slowing of CpG depletion in SARS-CoV-2 genomes merits further investigation and it should not be dismissed as a mere coincidence.

When this manuscript was in revision, the Omicron VOC emerged. Since we had analyzed CpG content of the other four VOCs, we also analyzed 5,436 full-length Omicron sequences (see Materials and Methods for details) and found that the Omicron variant had marginally higher CpG content compared with the sequences from the beginning of the pandemic (supplementary fig. 4, Supplementary Material online). An early report indicates that the Omicron variant may be less pathogenic and may be associated with lower rates of hospitalization (Maslo et al. 2021). Higher CpG content of the HIV-1 envelope gene has been linked to better clinical outcomes (Wasson et al. 2017). However, we believe that detailed analysis of sequences along with clinical outcomes and laboratory markers may be needed to understand the association, if any, between CpG content and the disease outcomes in SARS-CoV-2.

**Fig. 8.**
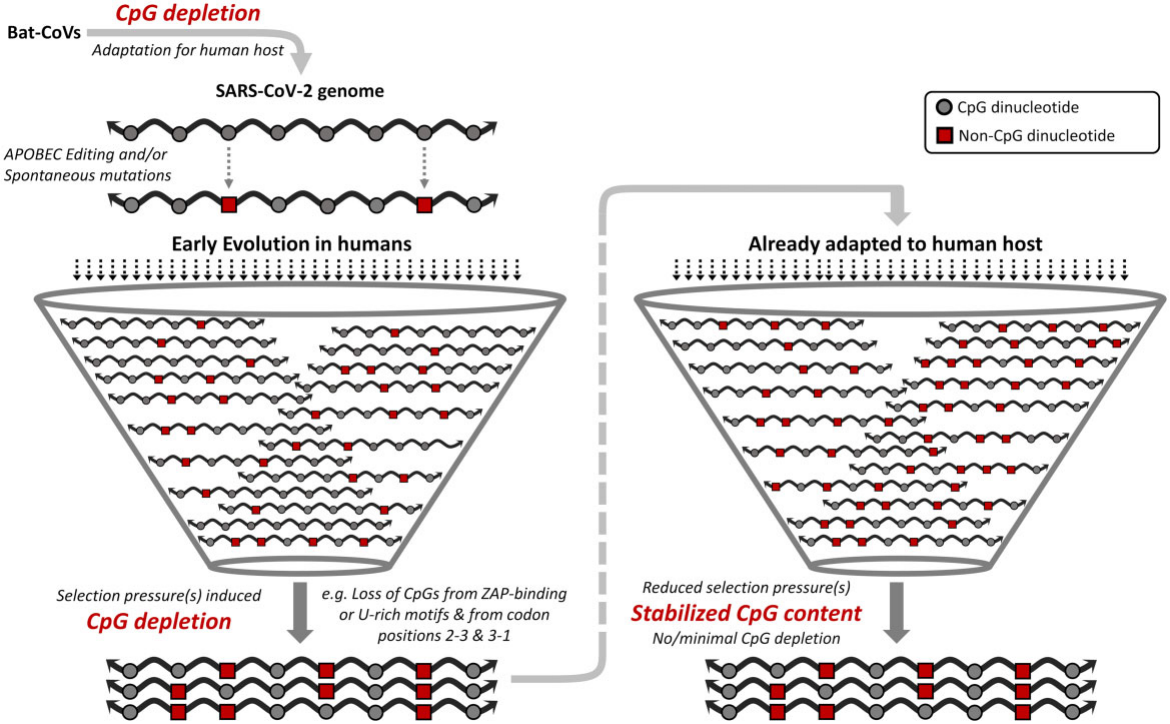
The changing dynamics of CpG depletion in SARS-CoV-2 genomes over time: a schematic representation highlighting the differences in the dynamics of CpG depletion during evolution of SARS-CoV-2 in humans. Adaptive evolution of bat coronaviruses has been linked to CpG depletion prior to infecting humans. The selection pressures leading to further CpG deletion during early evolution in humans are indicated. The CpG content of SARS-CoV-2 appears to have stabilized and reached an equilibrium following early evolution in humans. SARS, severe acute respiratory syndrome; CoV, coronavirus; ZAP, zinc-finger antiviral protein; APOBEC, apolipoprotein B mRNA-editing catalytic polypeptide.

The schematic in figure 8 highlights the depletion of CpGs in SARS-CoV-2 genomes during early evolution in humans followed by stabilization of CpG content during progressive adaptation to the human host.

In sum, we analyzed over 1.4 million full-length sequences of SARS-CoV-2 representing over 170 million human infections from January 2020 to May 2021. Our findings highlight: 1) that the extent of CpG depletion from SARS-CoV-2 genomes is modest (i.e., on average only about 2–3 CpGs are lost from the genome during this period), 2) that the rate of CpG depletion in SARS-CoV-2 genomes is highest during early evolution and it gradually decreases almost reaching an equilibrium with increasing number of infections and adaptation to human hosts, and 3) a role for specific selection pressures leading to the avoidance of CpGs in SARS-CoV-2 genomes. Analysis of the coding regions of SARS-CoV-2 suggests that CpG depletion is more pronounced at codon positions 2-3 and 3-1 than at codon position 1-2. We also identify that the loss of the terminal CpG from ZAP-binding motifs is the major driving force for the deselection of these motifs from the SARS-CoV-2 genomes. However, the extent of CpG depletion from within and outside the ZAP-binding motifs in SARS-CoV-2 genomes are comparable, indicating the existence of other evolutionary forces that shape the CpG content in virus genomes. Furthermore, we also demonstrate that SARS-CoV-2 genomes selectively lose CpG-motifs in a U-rich context; this may be a mechanism by which the virus minimizes stimulation of TLR7 which regulates key innate immune responses.

The alpha-, beta-, and delta-variant have lower CpG content compared with the sequences from the beginning of the pandemic. The lineage-defining substitutions in the gamma variant that lead to the net gain of two CpGs suggest that the advantages provided by these substitutions overweigh those provided by the deselection of CpGs. The timeline for the emergence of VOCs intriguingly appears to coincide with beginning of CpG content stabilization in SARS-CoV-2 genomes.

Importantly, our findings provide evidence that the rate of CpG depletion is not constant and varies temporally during virus adaptation to the host. We also demonstrate that CpG depletion rates are highest during early virus evolution in humans and progressive adaptations in the host are associated with slowing of CpG depletion from SARS-CoV-2 genomes. This work provides new insights on the fundamental temporal aspects of CpG depletion including the extent of CpG depletion and the dynamics of CpG depletion in SARS-CoV-2 genomes during evolution in human hosts.

## Materials and Methods

### Sequences

We retrieved all available SARS-CoV-2 full-length sequences (*n* = 1,612,859) in GISAID (https://www.gisaid.org/, last accessed January 16, 2022) (Shu and McCauley 2017) on August 24, 2021 for samples collected between January 1, 2020 and May 31, 2021 using filters to include sequences with high coverage sequences from human hosts. The following categories of sequences were excluded: 1) sequences less than 29,700 or more than 30,000 nucleotides, 2) sequences with ≥300 Ns (ambiguous base-positions), 3) entries from outside China before January 13, 2020, as there were no documented cases outside China in this period (Li et al. 2020), and 4) Sequences with a Z score ≥3 for nucleotide composition, GC content, dinucleotide odds ratio for CpG and GpC, and CpG numbers and percentages. In addition, the full-length sequences that were not included in the multiple sequence alignment (MSA) available in GISAID (as on September 6, 2021 for samples collected from January 1, 2020 to May 31, 2021) were also excluded. Applying these filters, we selected a total of 1,410,423 (Accession numbers are provided in supplementary table 1, Supplementary Material online) complete SARS-CoV-2 genomes for analysis. The SARS-CoV-2 WIV04 strain/isolate was used as the reference sequence (Accession ID: EPI_ISL_402124).

For analysis of VOCs, the aforementioned inclusion and exclusion criteria were applied on full-length SARS-CoV-2 sequences retrieved on August 22, 2021 for samples collected between January 1, 2020 and May 31, 2021. Sequences were classified as VOCs (i.e., alpha-, beta-, gamma-, and delta-variants) using the appropriate filter for variants in GISAID; the sequences that were not classified as a VOC in GISAID are referred to as non-VOCs in this manuscript. Outliers from each of the VOCs and non-VOCs were excluded, resulting in a total of 616,626 sequences for the alpha variant, 16,247 sequences for the beta variant, 34,162 sequences for the gamma variant, 32,109 sequences for the delta variant, and 717,950 sequences for non-VOCs (Accession numbers are provided in supplementary table 3, Supplementary Material online). Information on number of documented cases of SARS-CoV-2 infections were obtained from Our World in Data (https://ourworldindata.org/, last accessed October 1, 2021) (Ritchie et al. 2020). A total of 170,787,092 million cases were documented from January 1, 2020 to May 31, 2021.

The SARS-CoV-2 sequences analyzed in this study were from samples collected between January 1, 2020 and May 31, 2021, as described above. When this manuscript was in revision, the Omicron VOC emerged. Since we had analyzed the CpG content of all other VOCs in this manuscript (as they emerged before May 2021), we also wanted to analyze the CpG content of the Omicron variant. On January 16, 2022, we downloaded all available full-length Omicron sequences with high coverage from GISAID. After using the filters that we used for all other sequences in this study (as described above), we analyzed a total of 5,436 full-length Omicron sequences for CpG content (supplementary table 7, Supplementary Material online). No further analysis was done on the Omicron sequences.

### Calculations

A python code was used to extract information on the sequence length, date of sample collection, mononucleotide frequencies, and dinucleotide frequencies. The percentage frequencies for mono- and dinucleotides were calculated using the exact sequence length for a given sequence after excluding the Ns in the sequence. The dinucleotides *O*/*E* ratios were calculated using the formula:

(1) $${\left(O/E\right)}_{X_{p}Y}=\left[f\left(X_{p}Y\right)/f\left(X\right)f\left(Y\right)\right]\times G$$

where *f*(*X_p_Y*), observed frequency of dinucleotide; *f*(*X*), frequency of nucleotide X; *f*(*Y*), frequency of nucleotide Y; and *G*, genome length.

The MSA available in GISAID with a total of 1,410,423 full-length sequences as described above was used for analyzing the number of sequences that: 1) lost a CpG dinucleotide from each of the CpG sites (i.e., *n* = 439 CpG sites present in the SARS-CoV-2 WIV04 reference sequence), 2) lost CpGs from each gene (includes genes encoding nonstructural protein, structural proteins, and all annotated ORFs; annotation as per GISAID), 3) lost CpGs from each of the three codon positions in the SARS-CoV-2 coding region, and 4) lost CpGs from the terminal CpG sites within ZAP-binding motifs. A python script was used to map ZAP-binding motifs (i.e., C(n_*m*_)G(n)CG, where *m* = 4/5/6/7/8) and CpG sites by analyzing MSA for the 1,410,423 full-length sequences. ZAP-binding motifs and CpG sites in each sequence and their locations in the genome were analyzed using re module (v 2.2.1) in python. Similarly, we also identified CpGs in a A/U-rich context by mapping motifs flanked by A/U (i.e., (A/U)CG(A/U)) and mapping the U-content of five flanking bases (i.e., NNN-CG-NN). These (A/U)-rich motifs have been described previously (Greenbaum et al. 2009; Jimenez-Baranda et al. 2011). ZAP-binding motifs were excluded for analysis of CpG motifs in an (A/U)-rich context.

For line plots in figure 2, the minimum resolution in time for sequences analyzed is 1 day and since each day corresponds to many available sequences, mean value for individual days were calculated. We have data on the number of cases from January 22, 2020. However, there are a total 15 days in between January 1, 2020 and January 21, 2020 for which we have sequence data but no data on the number of infections. Thus, the average values for 52 sequences submitted until January 21, 2020 were analyzed and used as the baseline (day one). We had a total of 497 points (a point for each day) from January 21, 2020 to May 31, 2021. For each day of the study period, we plotted the CpG content of the sequences analyzed against the total number of documented infections in the world (cumulative) at the end of that day (blue line in fig. 2). For example, on August 9, 2020, we have 630 sequences which were averaged out for the metrics (e.g., CpG numbers; plotted on the left *Y* axis) and cumulative cases at the end of that day stands at 19.87 million (plotted on *X* axis). Further, for smoothening, we plotted a tandem, overlapping 10-unit (10-day) moving average on the same graph (orange line in fig. 2), along with 95% confidence interval of this moving average (green bands in fig. 2). The confidence intervals were calculated using the formula:

(2) $$\mathrm{C.I.}=\bar{x}\pm t_{9,0.025}\times \left(\frac{s}{\sqrt{n}}\right)$$

where, $\bar{x}$ is sample mean, $t_{9,0.025}$ is *t*-distribution 95% confidence value (=2.262), s is sample SD, and n is sample size. We then used the minimum–maximum value in the study period (to understand the extent of depletion) and scaled the difference between each value and the maximum value over this range. These scaled values were then multiplied by 100 to convert them into percentage depicting the extent of CpG depletion (fig. 2). The percentage depletion of CpG is calculated using the formula:

(3) $${\%\mathrm{depletion}}_{i}=\left(\frac{\max\left(x\right)-x_{i}}{\max\left(x\right)-\min\left(x\right)}\right)\times 100$$

where, i represents ith value and x represents per day averaged data. The percentage reduction in CpG content was plotted on the right *Y* axis (fig. 2), with *X* axis (cumulative number of infections on a given day) and *Y* axis on the left (CpG content) plotted as described above.

### Plots and Statistical Analysis

Python (*v*≥ 3.8.11), pandas (v 1.3.2), and NumPy (v 1.20.3) were used for data curation, data preprocessing, statistical analysis, and data visualization. Libraries/Packages in Python used for plots or statistical analyses are indicated in table 1.

**Table 1.** Libraries/Packages in Python Used for Plots or Statistical Analyses.

Library/Package	Plot/Statistical Test	Figures	Notes
Seaborn(v 0.11.2)	Line plot	Figure 1*a* and *b*; supplementary figures 2 and 3, Supplementary Material online	Percentages of mononucleotides and dinucleotides were plotted along a time axis of 1-month intervals. Since each month corresponds to several sequences, a band was plotted along with the mean value to depict the range of values in the 95% CI.
	Violin plot	Figures 1*c* and *d*, 3, 4*c*, 5*c* and *d*, 6, and 7; Supplementary figure 4, Supplementary Material online	The horizontal lines in the violin plots depict the lower quartile, the median, and the upper quartile.
	Box-and-Whisker plot	Figure 4*b*	The box shows the quartiles of the data set whereas the whiskers extend to show the rest of the distribution, except for points that are determined to be “outliers” using a function of the interquartile range.
SciPy.stats(v 1.7.1)	Mann–Whitney *U* test	Figures 1*c* and *d*, 3, 4*c*, 5*c* and *d*, 6, and 7; Supplementary figure 4, Supplementary Material online	*P* < 0.05 was considered significant.
	Kruskal–Wallis *H* test	Figure 7	*P* < 0.05 was considered significant.
Matplotlib(v 3.4.2)	Scatter plot	Figures 4*a* and 5*a* and *b*; Supplementary figure 1*b*, Supplementary Material online	In figure 4*a*, CpGs sites corresponding to the first 100 nt and last 100 nt of the reference sequence for SARS-CoV-2 were excluded from analysis to eliminate any potential bias caused by either increased number of Ns at the 5′ or 3′ ends.
Matplotlib(v 3.4.2)	Line plot	Figure 2 and supplementary figure 1, Supplementary Material online	See calculations for details.

## Supplementary Material

Supplementary data are available at *Molecular Biology and Evolution* online.

## Supplementary Material

msac029_Supplementary_Data
