# Supplementary material for: The Slowing Rate of CpG Depletion in SARS-CoV-2 Genomes Is Consistent with Adaptations to the Human Host
Source: Mol Biol Evol. 2022 Feb 3;39(3):msac029. doi: 10.1093/molbev/msac029 (PMC8892944; doi:10.1093/molbev/msac029)
Supplement: msac029_Supplementary_Data [file msac029_supplementary_data.zip › Additional file 1_Supplementary figures.pdf]

ADDITIONAL FILE 1

The slowing rate of CpG depletion in SARS-CoV-2 genomes is consistent with adaptations to the human host.

Akhil Kumar [a,1], Nishank Goyal [b,1], Nandhini Saranathan[a], Sonam Dhamija [c,d], Saurabh Saraswat [a], Manoj B. Menon[a] and Perumal Vivekanandan[a, #]

[a] Kusuma School of Biological Sciences, Indian Institute of Technology Delhi, New Delhi-110016, India

[b] Department of Chemical Engineering, Indian Institute of Technology Delhi, New Delhi-110016, India

[c] CSIR-Institute of Genomics and Integrative Biology, New Delhi-110025, India

[d] Academy of Scientific and Innovative Research (AcSIR), Ghaziabad- 201002, India

[1] These authors contributed equally.

[#] Corresponding Author. Email: vperumal@bioschool.iitd.ac.in ; Tel: 011-26597532; Fax: +91-11-26582037

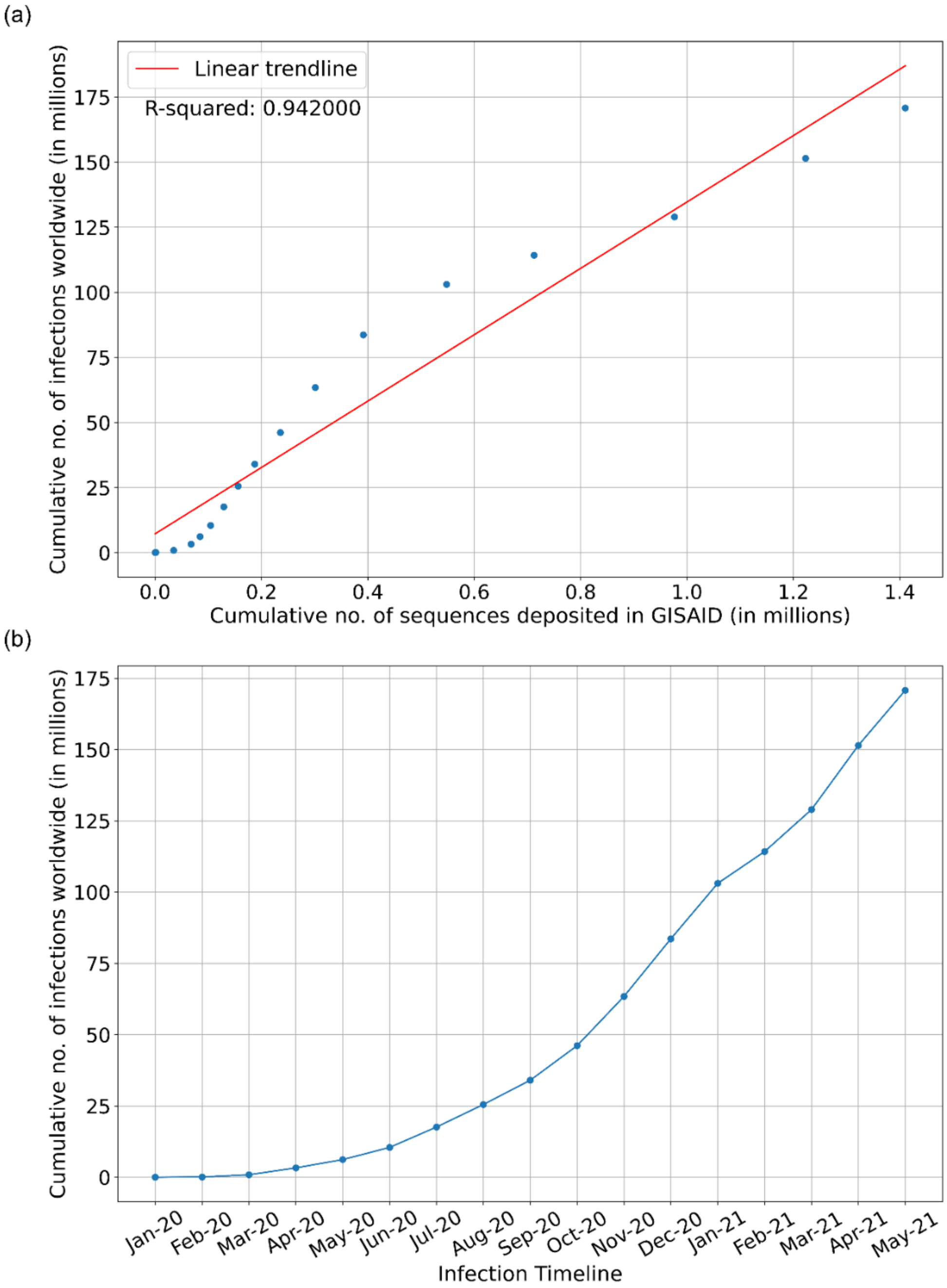

Supplementary figure 1

Documented SARS CoV-2 infections, sequences available in GISAID and the infection timeline.

(a) Scatter plot showing a strong correlation between the cumulative number of documented infections worldwide and the cumulative number of full-length SARS CoV-2 sequences available in GISAID. This finding suggests that the number of full-length SARS CoV-2 sequences available in the GISAID is proportional to the number of infections throughout the pandemic.

(b) A Scatter plot showing the cumulative number of documented infections increase exponentially with time from January 2020 to May 2021.

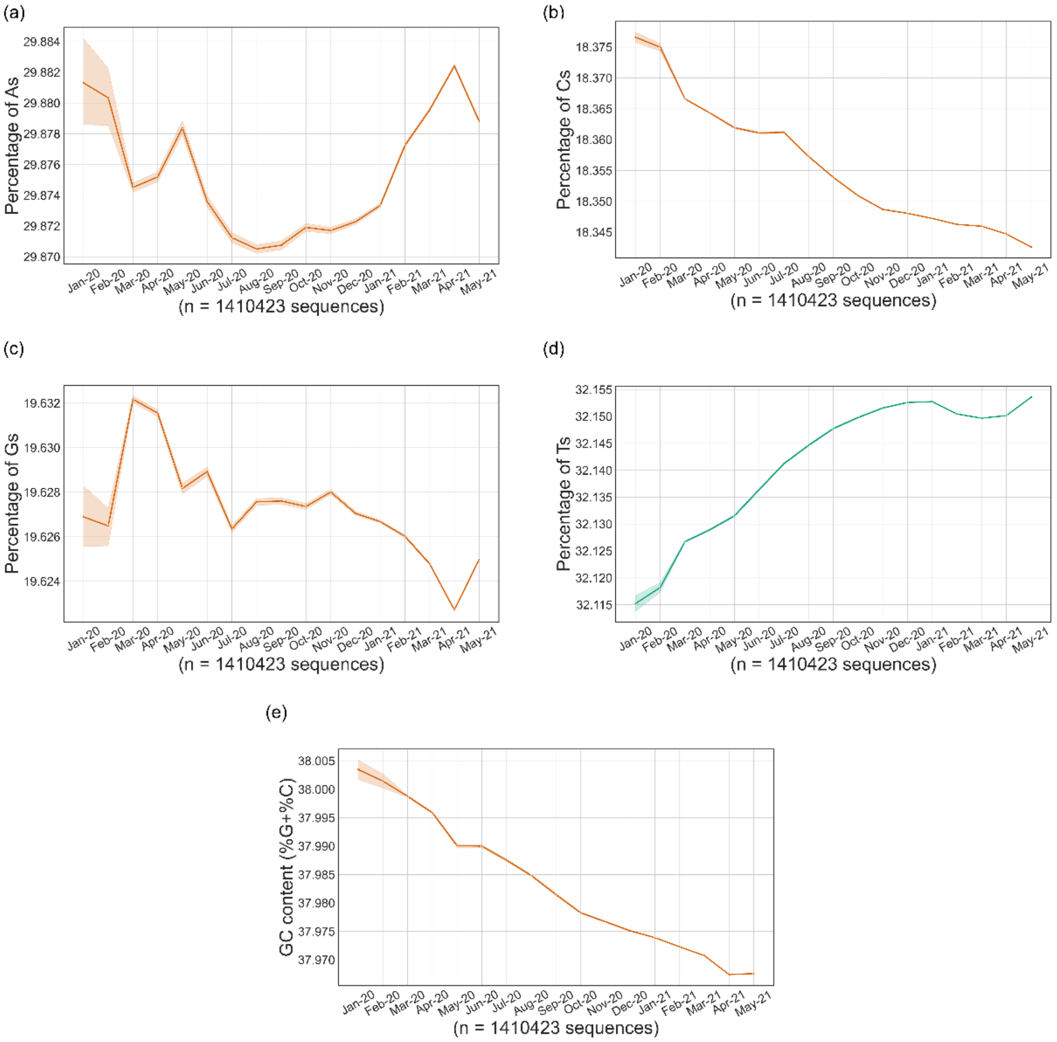

Supplementary figure 2

Analysis of mono-nucleotide composition:

Full-length sequences of SARS-CoV-2 were grouped month-wise based on the date of sample collection. Line graphs indicate the monthly mean of (A) Percentage of As (B) Percentage of Cs (C) Percentage Gs (D) Percentage Ts (E) GC Content

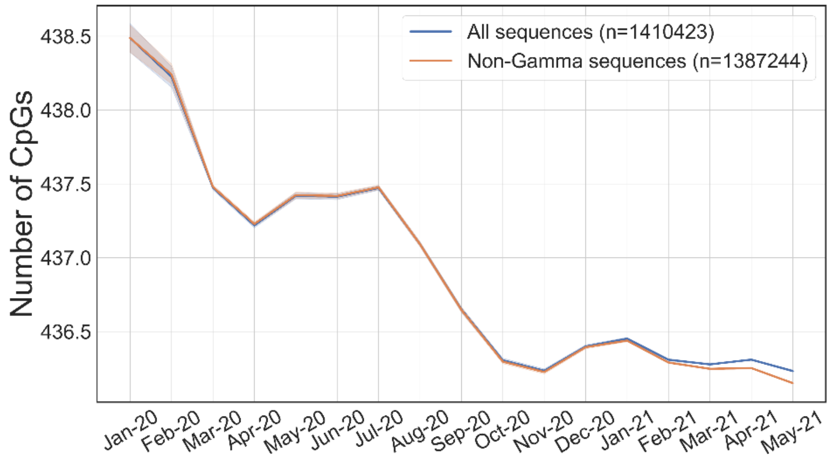

Supplementary Figure 3: High CpG content of SARS-CoV-2 gamma variant does not affect any of the conclusions on the extent of CpG depletion in early evolution and the subsequent stabilization of CpG content: The gamma variant has higher CpG content as compared to other VOCs and the sequences from the beginning of the pandemic (Figure 6). Temporal analysis of CpG content of SARS-CoV-2 genomes were re-analysed after excluding gamma sequences (<2.5% of all sequences).

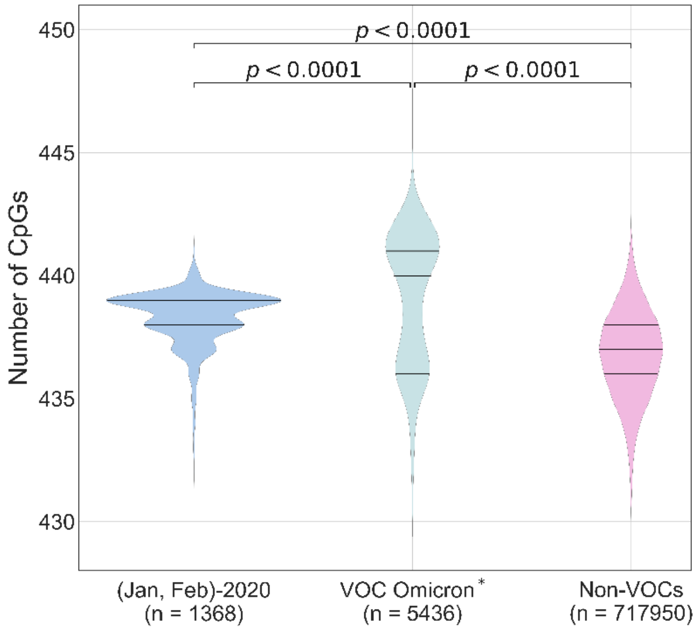

Supplementary Figure 4

CpG dinucleotide content of SARS-CoV-2 Omicron variant: Violin plots show the distributions of the content of CpG dinucleotides in the whole genomes of Omicron variant. The CpG content of sequences

from the beginning of the pandemic and non-VOCs from Figure 7 have been plotted for comparison. The CpG content of the Omicron variant is higher as compared to the sequences from the beginning of the pandemic and non-VOCs (P<0.0001). (Mann-Whitney U test). While sub-lineages are emerging among the Omicron variants, the substitutions in the Omicron sequence (Genbank accession ID OL672836.1, listed as B.1.1.529 lineage in https://viralzone.expasy.org/9556) have a gain of 4 CpGs and a loss of 3 CpGs. This finding may explain the higher CpG content among Omicron variants.
