# Supplementary material for: The Slowing Rate of CpG Depletion in SARS-CoV-2 Genomes Is Consistent with Adaptations to the Human Host
Source: Mol Biol Evol. 2022 Feb 3;39(3):msac029. doi: 10.1093/molbev/msac029 (PMC8892944; doi:10.1093/molbev/msac029)
Supplement: msac029_Supplementary_Data [file msac029_supplementary_data.zip › Author checklist.docx]

**Author Manuscript Submission Checklist (Original manuscripts only)**

Answer all the questions below, and then upload it as a supplementary file named (Author Checklist.doc).

Submitting Author’s Name- Dr.Vivekanandan Perumal

| (1) Did you read information about the [Manuscript Type](http://www.oxfordjournals.org/our_journals/molbev/manuscript_types.html) selected? | Yes |
| --- | --- |
| (2) Is the manuscript title no more than 100 characters (including spaces) and suitable for a wider audience? | Yes |
| (3) Did you use 1.5 line spacing throughout the manuscript with at least an 11 point font in the main text, figure legends, and references? | Yes |
| (4) Is the length of the main manuscript, including the text, figures, references, and tables no more than 30 pages? *Include other information in the supplementary files judiciously.* | Yes |
| (5) For first submissions, have you uploaded a single main manuscript file as a PDF that contains the main text, tables, figure captions, and figures? *Supplementary information is uploaded separately.* | N/A |
| (6) Is the manuscript arranged in order with proper headings for [Manuscript Type](http://www.oxfordjournals.org/our_journals/molbev/manuscript_types.html)? | Yes |
| (7) Are the: [*Supplementary files*](http://www.oxfordjournals.org/our_journals/molbev/supplementary_information.html) in the following format(s): Plain text (.txt); HTML (.html, htm); Jpeg (.jpg, .jpeg); GIF (.gif); QuickTime video (.mov); MPEG Movie (.mpg); MS_AVI Video (.avi); Adobe PDF (.pdf); MS Excel Spreadsheet (.xls) | Yes |
| (8) Have you read and followed the [General Author Guidelines](http://www.oxfordjournals.org/our_journals/molbev/general_author_guidelines.html) for MBE? | Yes |
| (9) For resubmissions allowed/invited by the editors, did you provide the previous manuscript ID #? | N/A |
