# Supplementary material for: The Slowing Rate of CpG Depletion in SARS-CoV-2 Genomes Is Consistent with Adaptations to the Human Host
Source: Mol Biol Evol. 2022 Feb 3;39(3):msac029. doi: 10.1093/molbev/msac029 (PMC8892944; doi:10.1093/molbev/msac029)
Supplement: msac029_Supplementary_Data [file msac029_supplementary_data.zip › Rebuttal.docx]

**REBUTTAL**

**REVIEWER 1**

**Major comments:**

1. In the text, the authors do an excellent job of highlighting that the loss of CpG dinucleotides is small—approximately 3 CpGs were lost out of >400 through May 2021. However, the Y-axis in the Figure 1 panels do not convey this point clearly since they are cropped at the bottom in an artificial way to make the small change look large. For example, in Figure 1A, the Y-axis looks like it is from 436 to 439, a range of 3 CpGs. For Figure 1A and 1C, the Y-axis should start at 0 to show the full range of the CpG abundance. If the authors want to highlight the trends shown in panels 1A and 1C, they can be included but shown as a subsection of the more relevant graph that goes from 0 to approximately 445 CpGs. Similarly, panels 1B and 1D should start at 0% to show that the change is modest and the current panels can be presented as a subsection of these plots. Since some of the initial papers on CpG abundance in SARS-CoV-2 implied that there was a substantial loss of CpGs, it is important to show the magnitude of change in CpG abundance clearly. Once this has been shown clearly in Figure 1, the presentation of the remaining figures showing CpG abundance, such as Figure 2, is fine.

**Response:** As suggested, the figures showing the full range starting from 0 have been included in Figures 1a and 1b of the revised manuscript.

2. The observation in Figure 4 is interesting in that only a limited number of CpG sites are lost in a substantial number of sequences. Can a supplementary data file be created that lists the conservation of each site so this can be used by others? Presumably this is the underlying data for Figure 4A?

**Response:** As suggested by the reviewer, the data on conservation of CpGs plotted in Figure 4A has been included as supplementary Table 6 in the revised manuscript.

3. For Figure 6, a recent paper compared the effect of ZAP on a SARS-CoV-2 isolate similar to the early pandemic strains and an isolate representing the alpha variant of concern (VOC) (Kmiec et al PLoS Pathog. 2021 Oct 25;17(10):e1009726). This paper found that the alpha VOC was restricted by ZAP as efficiently as the early pandemic isolate. This should be incorporated by the authors since it shows that the small decrease in CpG abundance in the alpha VOC does not affect its sensitivity to ZAP.

**Response:** As suggested, we have now included a line on the sensitivity of the alpha variant to ZAP along with the appropriate reference.

4. Figure 7 implies that CpG abundance in the early SARS-CoV-2 isolates were not well adapted to humans and subsequently evolved in humans. This is speculative since there have been only very small changes in the CpG abundance over the course of the pandemic and some of these changes may have been driven by synonymous changes in the variants of concern that have worldwide penetrance, such as initially the alpha VOC and now the delta VOC. This also omits the observations by MacLean et al (PLoS Biol. 2021 Mar 12;19(3):e3001115) that there was a potent depletion of CpG abundance early in the SARS-CoV-2 lineage, most likely in bats. Therefore, CpG abundance was suppressed before it entered humans due to an earlier adaptive event. This needs to be incorporated into the model in Figure 7. The idea that the CpG abundance had to be depleted below a specific threshold before variants of concern could emerge in humans does not seem likely and this should probably be removed from the manuscript. Other selective pressures on the virus such as emerging population level immunity, social distancing, mask wearing, and economic shutdowns that limited the number of person-to-person contacts are more likely explanations for the	VOC	evolution.

**Response:** Figure 7 (Figure 8 in the revised manuscript) has been modified to include the adaptive CpG depletion in bats, as suggested by the reviewer along with the appropriate citation in the text. The speculation on emergence of VOCs after CpG stabilization has been removed from Figure 7 (Figure 8 in the revised manuscript) as suggested.

**Minor	comments:**
1. Line 50, page 6: Most APOBEC proteins deaminate ssDNA, not ssRNA

**Response:** The authors agree that most APOBECs deaminate ssDNA. However, said sentence refers to the C-to-U deamination which is specific to RNA. The sentence reads “APOBECs have been linked to C-to-U deamination of bases in single-stranded RNA”.

2. For Figure 3, the statistical test used should be in the figure legend.

**Response**: The authors thank the reviewer for this suggestion, the statistical test used (Mann-Whitney U test) is now indicated in the figure legend.

**Reviewer 2
Comments to the Author**
The authors have examined the question of whether the CpG changes in SARS-CoV-2 during the first years of the pandemic has changed appreciably or is indicative of selection. It is an interesting question. As the authors note, the most comparable case history we have is the evolution influenza, where CpG depleted over time, coming to an equilibrium with their host. In the case of the HIV pandemic the CpG levels were already quite low reflecting a host that is closer to humans. Early analysis of SARS-CoV-2 (SCV2) have shown that compared to other non-human coronaviruses its CpG levels were quite low, suggesting there is not much of a selective pressure. The authors therefore took advantage of the uniquely rich dataset around COVID-19 to see if there was any evidence of evolutionary change, as had been indicated as possible in earlier analyses (also published in MBE). The analysis is excellent, well-written and thorough and is ready for publication. I have only a few questions:

• The authors note an inconsistency that makes their argument slightly confusing in places. The loss in CpG number is slight, yet the trend is clear and given the large numbers of SCV2 sequences, highly significant. Can the authors plots the variance as a function of time?

**Response:** In the figure below, we have plotted the variance in CpG numbers and percentage as a function of time. In addition, we have also plotted the coefficient of variation against time. A subtle increase in the variance and the coefficient of variation is expected and it may indicate heterogeneity of sequences with time, emergence of VOCs and the circulation of multiple variants at a given time. Nonetheless, the trend line plot in Figures 1 and 2 in the manuscript and the violin plots in Figure 1, clearly capture the modest but consistent and significant reduction in CpGs.

**Figure:** Line graphs showing an increasing trend in variance and coefficient of variation of the percentage of CpGs (a and c) and numbers of CpG (b and d) during SARS-CoV-2 evolution in humans.

• It would also be useful to add Omicron to the analysis. Omicron also seems to be milder so that association would be interesting.

**Response:** We had originally analysed sequences available up to February 2021. Based on the request from reviewers to include additional sequences, we had included nearly a million new sequences and did all the analysis in the manuscript and submitted a revised version in October 2021. Omicron did not exist when we submitted the revision. However, based on the reviewer’s suggestion, we have now analysed the CpG content of Omicron sequences available in GISAID. Omicron sequences had marginally higher CpG content compared to the sequences from the beginning of the pandemic. This data is shown is shown in Supplementary Figure 4 of the revised manuscript.

• Its worth noting that ZAP is not the only mechanism implicated in CpG loss (though it's the most well validated). Earlier work (Jimeneza Baranda, et al, J Virol, 2011), implicated that TLR 7 or 8 might sense CpGs in (A/U) rich contexts. If one excludes the ZAP associated CpGs does one see a trend?

**Response:** We sincerely thank the reviewer for this suggestion. We excluded ZAP motifs and analyzed extent of CpGs lost from A/U rich context based on (Jimenez-Baranda et al. 2011). In addition, we also analysed the CpG motifs flanked by A/U [i.e. (A/U)CG(A/U)] as described by (Greenbaum et al. 2009). The extent of CpG loss was not influenced for CpGs motifs flanked by A/U. Nonetheless, as proposed by the reviewer, CpG loss in SARS-CoV-2 genomes was more pronounced in CpGs from a U-rich context (i.e 4Us in the motif NNN-CG-NN). This data has been included in Figure 6 of the revised manuscript; the results have been discussed in the text.

• As the authors mention, SCV2 may be closer to equilibrium and therefore not under as much pressure as an avian virus to lose CpGs. The Greenbaum et al 2014 paper calculated a rate of CpG loss for influenza. Do the authors think that current strains have reached an equilibrium? Do they have similar CpG content to Flu B or the human genome for instance?

**Response:** The CpG O/E for the human genome is approximately 0.25 (Saxonov et al. 2005) and that for the CDS in the human genome is about 0.47 (Cheng et al. 2013). The range of CpG O/E ratios range from 0.15 to 0.80 for RNA viruses infecting humans (Cheng et al. 2013) indicating that equilibrium may be reached at different CpG contents for different viruses. The CpG O/E ratio for Influenza B viruses is about 0.33 (Cheng et al. 2013) and that for SARS-CoV-2 is about 0.40.

Nonetheless, our argument that SARS-CoV-2 may be closer to equilibrium in terms of CpG loss is based on tangible CpG loss during evolution humans during the first 40-50 million infections and the absence of additional CpG loss in the subsequent 120 million documented human infections.

REFERENCES

Cheng X, Virk N, Chen W, Ji Shuqin, Ji Shuxian, Sun Y, Wu X. 2013. CpG Usage in RNA Viruses: Data and Hypotheses. PLoS ONE. 8(9). doi:10.1371/journal.pone.0074109.

Greenbaum BD, Rabadan R, Levine AJ. 2009. Patterns of oligonucleotide sequences in viral and host cell RNA identify mediators of the host innate immune system. PLoS ONE. 4(6). doi:10.1371/journal.pone.0005969.

Jimenez-Baranda S, Greenbaum B, Manches O, Handler J, Rabadan R, Levine A, Bhardwaj N. 2011. Oligonucleotide Motifs That Disappear during the Evolution of Influenza Virus in Humans Increase Alpha Interferon Secretion by Plasmacytoid Dendritic Cells. Journal of Virology. 85(8):3893–3904. doi:10.1128/jvi.01908-10.

Saxonov S, Berg P, Brutlag DL. 2005. A genome-wide analysis of CpG dinucleotides in the human genome distinguishes two distinct classes of promoters. *Proc. Natl. Acad. Sci USA.* 103(5):1412-17 [www.pnas.orgcgi](http://www.pnas.orgcgi) doi10.1073pnas.0510310103.
